# Impact of patent foramen ovale on short-term outcomes in children with biliary atresia undergoing living donor liver transplantation: a retrospective cohort study

**DOI:** 10.1186/s12871-023-02268-w

**Published:** 2023-09-15

**Authors:** Yuli Wu, Yongle Jing, Tianying Li, Lu Che, Mingwei Sheng, Lili Jia, Hongxia Li, Wenli Yu, Yiqi Weng

**Affiliations:** 1 Department of Anesthesiology, Tianjin First Central Hospital, 300192 Tianjin, China; 2 Department of Cardiology, Tianjin First Central Hospital, 300192 Tianjin, China; 3 School of Medicine, Nankai University, 300071 Tianjin, China

**Keywords:** Patent foramen ovale, Living donor liver transplantation, Biliary atresia, Complication

## Abstract

**Objective:**

To investigate the impact of patent foramen ovale (PFO) on the short-term outcomes of living donor liver transplantation (LDLT) in children with biliary atresia.

**Methods:**

With the approval of the hospital ethics committee, 304 children with biliary atresia who underwent LDLT in our center from January 2020 to December 2021 were enrolled. According to the results of echocardiography before the operation, the subjects were divided into the PFO group (n = 73) and the NoPFO group (n = 231). The baseline characteristics; intraoperative recipient-related data and donor-related data; incidence of postreperfusion syndrome (PRS); postoperative mechanical ventilation time; ICU stay duration; postoperative hospital stay duration; liver function index; incidences of postoperative complications including acute renal injury (AKI), graft dysfunction, hepatic artery thrombosis (HAT) and portal vein thrombosis (PVT); and one-year survival rate were compared between the two groups.

**Results:**

The median age in the PFO group was 6 months and that in the NoPFO group was 9 months (*P* < 0.001), and the median height (65 cm) and weight (6.5 kg) in the PFO group were significantly lower than those in the NoPFO group (68 cm, 8.0 kg) (*P* < 0.001). The preoperative total bilirubin level (247 vs. 202 umol/L, *P* = 0.007) and pediatric end-stage liver disease (PELD) score (21 vs. 16, *P* = 0.001) in the PFO group were higher than those in the NoPFO group. There were no significant differences in the intraoperative PRS incidence (46.6% vs. 42.4%, *P* = 0.533 ), postoperative mechanical ventilation time (184 vs. 220 min, *P* = 0.533), ICU stay duration (3.0 vs. 2.5 d, *P* = 0.267), postoperative hospital stay duration (22 vs. 21 d, *P* = 0.138), AKI incidence (19.2% vs. 24.7%, *P* = 0.333), graft dysfunction incidence (11.0% vs. 12.6%, *P* = 0.716), HAT incidence (5.5% vs. 4.8%, *P* = 0.762), PVT incidence (2.7% vs. 2.2%, *P* = 0.675) or one-year survival rate (94.5% vs. 95.7%, *P* = 0.929) between the two groups.

**Conclusion:**

The presence of PFO has no negative impact on short-term outcomes in children with biliary atresia after LDLT.

## Introduction

The foramen ovale exists in fetuses with normal circulation. During the embryonic period, oxygen-enriched blood is able to flow directly from the right atrium to the left atrium, and the left atrial shunt blood is mixed with a small amount of blood from the pulmonary vein and flows into the left ventricle and ascending aorta [1]. After birth, the increase in left atrial pressure and the decrease in pulmonary vascular resistance promote the spontaneous closure of the foramen ovale. Under normal circumstances, the foramen ovale closes gradually within one year after birth, but foramen ovale closure is complete by the age of 2 years in 75% of people [2–5]. Due to the existence of intracardiac channels, patent foramen ovale (PFO) can lead to abnormal embolism. Among adult patients undergoing major noncardiac surgery, PFO is associated with an increased risk of perioperative ischemic stroke, in-hospital mortality, and rehospitalization within 30 days after surgery [6]. There have been few related studies in pediatric patients. To date, there have been no studies on the incidence of PFO in children with biliary atresia in living donor liver transplantation (LDLT). The effect of PFO on the outcomes in children undergoing LDLT remains to be investigated.

## Materials and methods

### Participants

Following approval by the hospital ethics committee (No.2022DZX02), pediatric patients (age < 18 years) with biliary atresia who underwent LDLT in Tianjin First Central Hospital from January 2020 to December 2021 were included in this study.

Exclusion criteria included retransplantation, pediatric patients with metabolic disease, Alagille syndrome, Langerhans cell hyperplasia and portal vein cavernous transformation, and incomplete information recorded before and during surgery.

### Echocardiography assessment

Transthoracic two-dimensional echocardiography (Philips, IE33) was routinely performed in children undergoing LDLT. To capture the correct imaging plane, the probe was placed perpendicular to the atrial septum, the presence of an interatrial defect in 2-D or interatrial color flow signal in color flow doppler were highly suggestive of the presence of a PFO. If PFO was detected by transthoracic echocardiography, the diameter was measured and recorded. Transplant surgeons, pediatricians and anesthesiologists jointly perioperatively managed the children.

### Anesthesia protocol

The children arrived without having received any medications and were monitored using ECG, pulse oximetry, and noninvasive monitoring techniques. Anesthesia was induced with midazolam (0.15 mg/kg), propofol (2–3 mg/kg), fentanyl (2–5 µg/kg), and rocuronium (0.6–1.0 mg/kg). After intubation, mechanical ventilation was performed with a fraction of inspired oxygen (FiO2) of 50-60%, a tidal volume of 8–10 ml/kg, a respiratory rate of 20–28/min, an inspiration-to-expiration ratio of (1.0:1.5)-2.0, and an end-tidal CO_2_ partial pressure of 30–35 mmHg. Maintenance anesthesia was performed with sevoflurane (1.5–2.5%), the intravenous infusion of propofol (9–15 mg/kg/h), intermittent intravenous fentanyl administration (1–3 µg/kg), and the intravenous infusion of atracurium besylate (1–2 µg/kg/min). Right internal jugular vein puncture was performed under ultrasound guidance to monitor central venous pressure (CVP). A radial artery puncture was performed as an invasive means of monitoring arterial pressure.

Albumin and acetate Ringer’s solution were used for fluid therapy based on the hemodynamic parameters and CVP. Red blood cells (RBCs) were given to maintain a hemoglobin level of 8–10 g/dL. Coagulation function was detected by a Sonoclot analyzer (Sienco, Inc., Arvada, CO, USA). When there was an obvious coagulation disorder, fresh frozen plasma (FFP) was infused. The patients were monitored, and fluctuations in systolic blood pressure and heart rate during surgery were maintained within 20% of the baseline values. When hemodynamic changes occurred, we used anesthetics, cardioactive drugs, and fluids in any necessary interventions.

### Surgical procedure

For the donor, a left lobectomy was performed, and piggyback liver transplantation was performed for the recipient. HTK (histidine-tryptophan-ketoglutarate) solution was used as a perfusion solution to perfuse the transplanted liver. After occlusion of the inferior vena cava (IVC), the left hepatic vein of the transplanted liver was anastomosed with the recipient hepatic vein, and the inferior vena cava was opened after the anastomosis completed. The donor and recipient portal veins were anastomosed. Reperfusion of the liver graft started with opening of the portal vein. The venovenous bypass was not performed during the operation. The left hepatic artery of the donor was anastomosed with the hepatic artery of the recipient. After arterial reperfusion, the bile duct was connected to the recipient’s bile duct (choledocho-choledochostomy) or to a small bowel loop (hepaticojejunostomy). The vascular morphology and blood flow velocity were examined by ultrasound after hepatic artery opening and abdominal closure, respectively.

### Statistical analysis

Children who died were followed up until the date of death, and the rest of the recipients were followed up until December 31, 2022. All continuous variable data were tested for normality. The measurement data with normal distributions are expressed as the mean ± standard deviation, and independent sample t tests were used to compare the two groups; non-normally distributed continuous variables are expressed as the median (interquartile range), and the comparisons between groups were performed with two independent samples nonparametric tests. Classification variables are expressed as the number of cases and percentages, and chi-square tests (Pearson chi-square tests) or Fisher’s exact tests were used for analysis. A Kaplan Meier survival curve was used to describe the survival of the patients. SPSS software version 20.0 (SPSS, Inc., Chicago, IL, USA) was used for statistical analysis. A value of *P* < 0.05 was considered to indicate a significant difference.

## Results

**Fig. 1:**
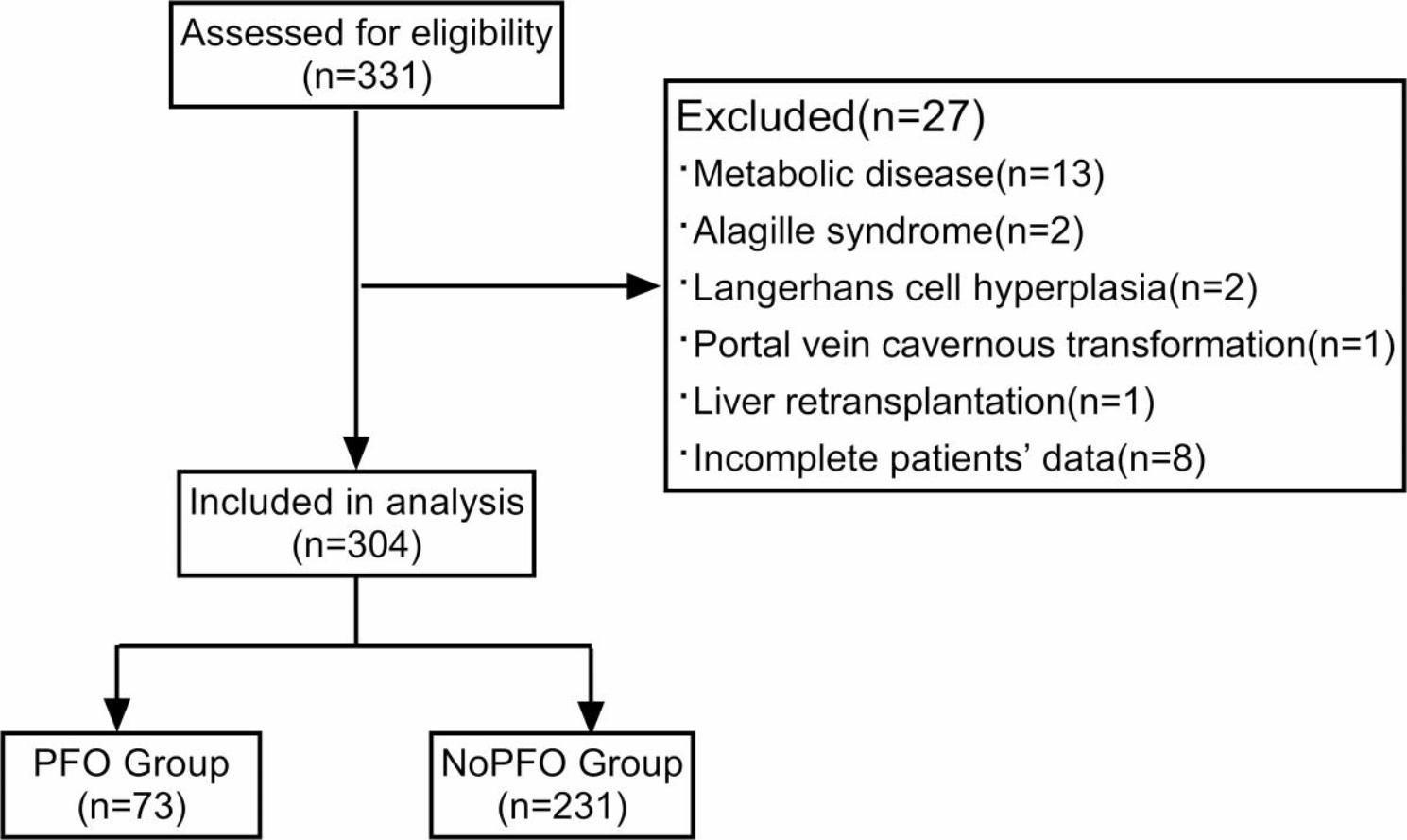
Flow chart of patients

A total of 331 children underwent LDLT in Tianjin First Central Hospital from January 2020 to December 2021 (Fig. 1). Of these, 27 patients were excluded from the study, including 13 patients due to metabolic disease, 2 patients due to Alagille syndrome, 2 patients due to Langerhans cell hyperplasia, 1 patient due to portal vein cavernous transformation, 1 patient due to transplant liver failure and 8 patients due to incomplete data. Finally, a total of 304 children with biliary atresia were included, of whom 73 patients (24%) were diagnosed with PFO by echocardiography and were included in the PFO group. No PFO was found in the NoPFO group (n = 231).

Table 1 summarizes the baseline characteristics of the children, including age, male gender, height, weight, PELD score, left ventricular ejection fraction (LVEF), ALT, AST, total bilirubin, international normalized ratio (INR), creatinine, and hemoglobin. The median age of the children with PFO was younger than that of those in the NoPFO group (*P* < 0.001). The median height (65 vs. 68 cm) and median weight (6.5 vs. 8.0 kg) were lower in the PFO group (*P* < 0.001). At the same time, the PFO group had a higher total bilirubin level (247 vs. 202 µmol/L, *P* = 0.007) and PELD score (21 vs. 16, *P* = 0.001). The median (interquartile range) diameter of PFO was 3.0 (2.5–3.3) mm in the PFO group. The male gender, LVEF, ALT, AST, INR, creatinine and hemoglobin levels in the two groups were similar, and there were no significant differences between the two groups (*P* > 0.05).

**Table 1:** Preoperative recipient-related data

Variable	PFO (n = 73)	NoPFO (n = 231)	*P*-Value
**Age (m)**	6(6–8)	9(6–15)	< 0.001
**Male gender (%)**	32(43.8%)	119(51.5%)	0.253
**Height (cm)**	65(61–68)	68(64–80)	< 0.001
**Weight (kg)**	6.5(6.0–7.0)	8.0(6.6–10.8)	< 0.001
**PELD score**	21(13–27)	16(5–23)	0.001
**LVEF (%)**	65(61–67)	64(62–67)	0.773
**ALT (U/L)**	95(71–175)	104(63–168)	0.861
**AST (U/L)**	208(122–336)	185(118–292)	0.241
**Total Bilirubin (µmol/L)**	247(147–352)	202(65–307)	0.007
**INR**	1.41(1.19–1.92)	1.37(1.12–1.78)	0.136
**Creatinine (µmol/L)**	13.0(12.0–15.0)	14.0(11.0-16.5)	0.538
**Hemoglobin (g/dL)**	92.4 ± 15.1	93.0 ± 16.9	0.79

LVEF, left ventricular ejection fraction; PELD, pediatric end-stage liver disease; ALT, alanine aminotransferase; AST, aspartate aminotransferase; INR, international normalized ratio

Analysis of the intraoperative data (Table 2) showed that there was no significant difference in the incidence of postreperfusion syndrome (PRS) between the two groups (46.6% vs. 42.4%, *P* = 0.533). In addition, there was no significant difference in the HR, MAP, CVP, or body temperature immediately before reperfusion between children with and without PFO, and there was no significant difference in the duration of anhepatic period, total operation time, total anesthesia time, intraoperative blood loss, the need for erythrocyte transfusion, the need for FFP transfusion or intraoperative urine volume between the two groups at the end of the operation. There was no significant difference in the cold ischemia time (85 vs. 86 min, *P* = 0.762) or graft weight (231 vs. 249 g, *P* = 0.175) of donors between the two groups.

**Table 2:** Intraoperative recipient-related data and donor-related data

Variable	PFO (n = 73)	NoPFO (n = 231)	*P*-Value
Vital signs immediately before reperfusion			
HR (beats/min)	118.0 ± 12.1	116.9 ± 13.3	0.541
MAP (mmHg)	58.1 ± 9.5	59.7 ± 10.8	0.276
CVP (mmHg)	5.7 ± 3.0	5.3 ± 2.7	0.321
Temperature (°C)	36.51 ± 0.75	36.45 ± 0.82	0.558
**Duration of anhepatic period (min)**	50(39–57)	48(40–59)	0.776
**PRS (%)**	34(46.6%)	98(42.4%)	0.533
**Donor-related data**			
Cold ischemic time (min)	85(66–105)	86(67–112)	0.762
Graft weight (g)	231(212–274)	249(215–279)	0.175
**At the end of surgery**			
Duration of surgery (min)	530(475–575)	550(495–600)	0.061
Duration of anesthesia (min)	612(550–640)	615(560–655)	0.497
Blood loss (ml)	300(200–400)	300(200–400)	0.339
Urine output (ml)	400(250–600)	400(280–600)	0.816
RBCs transfusion (units)	2.0(2.0–3.0)	2.0(1.5-3.0)	0.541
FFP (ml)	0(0-150)	0(0-150)	0.812

HR, heart rate; MAP, mean arterial pressure; CVP, central venous pressure; RBCs, red blood cells; FFP, fresh frozen plasma

The ventilation time; the postoperative ICU stay; the postoperative hospital stay; incidences of postoperative complications including acute renal injury (AKI), graft dysfunction, hepatic artery thrombosis (HAT) and portal vein thrombosis (PVT); the peak values of AST, ALT, and total bilirubin during the first 5 days after LDLT; and the one year survival rate were shown in Table 3. The mechanical ventilation time (184 vs. 220 min, *P* = 0.533), the postoperative ICU stay duration (3.0 vs. 2.5 d, *P* = 0.267), the postoperative hospital stay (22 vs. 21 d, *P* = 0.138), the incidence of AKI (19.2% vs. 24.7%, *P* = 0.333), graft dysfunction (11.0% vs. 12.6%, *P* = 0.716), HAT (5.5% vs. 4.8%, *P* = 0.762), and PVT (2.7% vs. 2.2%, *P* = 0.675) in the PFO group were similar to those in the NoPFO group. In the evaluation of liver function, there were no significant differences in the peak values of ALT, AST and total bilirubin during the first 5 days after LDLT between the two groups (*P* > 0.05). No patient had ischemic stroke in the two groups.

**Table 3:** Postoperative recipient-related data

Variable	PFO (n = 73)	NoPFO (n = 231)	*P*-Value
**Ventilation time (min)**	184(151–286)	220(133–352)	0.533
**Postoperative ICU stay (d)**	3.0(2.0–3.0)	2.5(2.0–3.0)	0.267
**Postoperative hospital stay (d)**	22(18–27)	21(16–27)	0.138
**AKI (%)**	14(19.2%)	57(24.7%)	0.333
**Graft dysfunction (%)**	8(11.0%)	29(12.6%)	0.716
**HAT (%)**	4(5.5%)	11(4.8%)	0.762
**PVT (%)**	2(2.7%)	5(2.2%)	0.675
**Liver function test during the first 5 days after LDLT**			
Peak ALT (U/L)	623(450–1142)	585(384–1082)	0.281
Peak AST (U/L)	700(538–1410)	692(466–1194)	0.192
Peak Total Bilirubin (µmol/L)	87(60–114)	83(56–121)	0.73
**One-year survival rate (%)**	69(94.5%)	221(95.7%)	0.929

ICU, intensive care unit; AKI, acute kidney injury; HAT, hepatic artery thrombosis; PVT, portal vein thrombosis; ALT, alanine aminotransferase; AST, aspartate aminotransferase

**Fig. 2:**
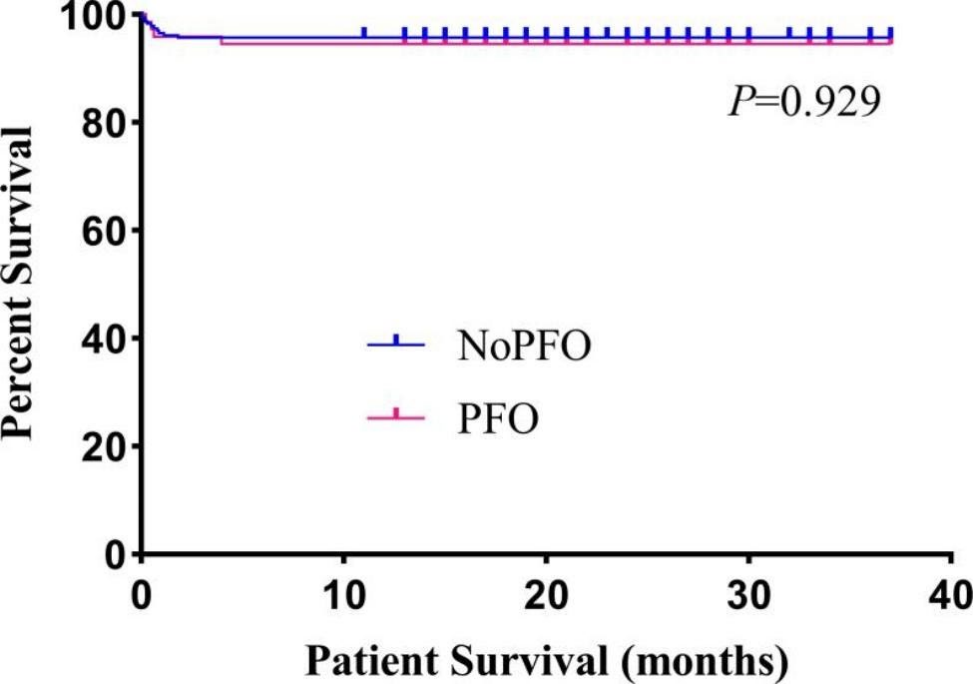
Comparison of the one-year survival rate after living donor liver transplantation between the two groups. PFO, patent foramen ovale

As shown in Fig. 2, the 1-year survival rates of the two groups were similar (94.5% vs. 95.7%, *P* = 0.929). All surviving children were discharged from the hospital. A total of 14 children died, 4 of whom were in the PFO group. The main causes of death were septic shock (2 patients), transplant liver failure (1 patient), and diffuse large B-cell lymphoma (1 patient). In the NoPFO group, 10 children died. The causes of death were multiple organ failure (3 patients), hemorrhagic shock (2 patients), lymphoproliferative disease (2 patients), transplant liver failure (1 patient), septic shock (1 patient) and vomiting asphyxia (1 patient).

## Discussion

The foramen ovale is the channel that connects the left and right atria in the fetus. At birth, with the first breath and consequent lung dilatation, the pulmonary blood flow increases, resulting in increased pressure in the left atrium, which functionally closes the foramen ovale. The anatomical closure of the foramen ovale is gradual and incomplete, and it remains open in up to 20–25% of adults [7–9]. Transthoracic echocardiography is performed routinely before LDLT in our center to determine whether the children have PFO. A common method of detecting PFO is transthoracic echocardiography (TTE) due to its easy availability, low cost and noninvasive nature. TTE helps identify the precise location (usually located at the cranial margin of the oval fossa) and anatomical variations (fissure, tunnel, aneurysm or windowing) of PFO. Studies have shown that when using the harmonic imaging mode, the sensitivity of TTE for diagnosing PFO is 91%, and the specificity is 93%. Harmonic imaging is a commonly used echocardiography technique in which ultrasonic signals are received at twice the transmission frequency. Second harmonic imaging can improve the quality of transthoracic two-dimensional images. Compared with basic contrast TTE imaging, second harmonic imaging can facilitate a more accurate diagnosis of PFO [10, 11]. According to the results of preoperative echocardiography, 73 of the 304 children in the study were diagnosed with PFO, with an incidence of 24%. PFO with biliary atresia is not uncommon in children undergoing liver transplantation.

Whether PFO causes an increase in perioperative complications or mortality in LDLT is the focus of our study. PRS is an inevitable physiological and pathological process in patients undergoing liver transplantation. It is usually defined as a mean arterial pressure 5 min after liver transplantation that is more than 30% lower than that before portal vein opening that persists for longer than 1 min [12]. This study followed the diagnostic criteria. PRS is usually accompanied by severe hemodynamic changes, such as decreased blood pressure, slow heart rate, arrhythmia, and even cardiac arrest in severe cases. PRS-related refractory hypotension can directly lead to poor liver reperfusion, seriously affect the recovery of function in the transplanted liver, lead to circulatory failure and even death, and directly affect the success or failure of liver transplantation. In this study, there was no significant difference in the incidence of PRS between the PFO group and the NoPFO group, indicating that PFO did not increase the incidence of this important adverse event after liver transplantation.

Also, AKI is a common complication after liver transplantation (LT). Although the exact pathophysiological mechanism of AKI is not fully understood, several studies have sought to explore the incidence and risk factors of AKI after LT. The study by Chan et al. in 2022 showed that the incidence of AKI in the first week after LDLT was 35.0%, and AKI was an independent risk factor for the incidence of major cardiovascular adverse events (MACE) and major renal adverse events (MAKE) [13]. The risk factors of AKI after LT included intraoperative hypotension, PRS, major bleeding, large RBC transfusion, and FFP infusion [14, 15]. Based on the Kidney Disease: Improving Global Outcomes (KDIGO) criteria [16], our result showed that the incidence of AKI was 23.4%. There were no significant differences in vital signs immediately before reperfusion, the incidence of PRS, blood loss, RBC transfusion and FFP infusion between the two groups, which may be the reason why there was no significant difference in the incidence of AKI between the two groups. Graft dysfunction is a common and serious problem that can occur after LT and poses significant challenges for both doctors and patients. The occurrence of graft dysfunction is approximately 25%, and it may differ between different medical centers, ranging from 10.8 to 36.3%, depending on the specific diagnostic criteria used [17]. The diagnostic criteria for postoperative graft dysfunction in this study were primarily based on the research conducted by Olthoff et al. [18]. Our results showed that there was no significant difference in the incidence of graft dysfunction between the PFO group and the NoPFO group (11.0% vs. 12.6%, *P* = 0.716). Donor age and body mass index, the degree of fatty liver, and Model for End-Stage Liver Disease (MELD) score were risk factors for graft dysfunction after LT [18, 19]. There is currently no evidence to support a connection between graft dysfunction and PFO.

Studies on adult patients with PFO have shown that there is a correlation between an unclosed foramen ovale and incident ischemic stroke, and the prevalence of PFO in patients with cryptogenic stroke is significantly higher than that in the general population [20, 21]. A large-sample multicenter study published in JAMA in 2018 showed that a preoperative diagnosis of PFO was significantly associated with an increased risk of perioperative ischemic stroke within 30 days after surgery in adult patients undergoing noncardiac surgery [22]. Therefore, some researchers suggest that high-risk patients should be screened for PFO before surgery to reduce the risk of abnormal embolism [23]. There is no clear consensus on whether it is necessary to actively close PFO before surgery to reduce the incidence of related complications in pediatric patients undergoing LT. Generally, PFO has little effect on cardiac hemodynamics and can remain untreated, and routine PFO screening in asymptomatic children is not recommended [24]. Therefore, the general view is that the management of PFO should be based on clear indications (such as significant hemodynamic changes or associated right ventricular overload) and that PFO occlusion is not recommended for the primary prevention of stroke in children [25]. In 2007, Allan et al. conducted a study that included 23 children with congenital heart disease (CHD) and end-stage liver disease (ESLD) who successfully received LDLT, and they found that structural heart defects associated with stable hemodynamics had no negative effects on the outcome of transplantation [26]. It was reported that in 14 children with PFO and ESLD, 43% of the PFO cases closed spontaneously in the early stage of transplantation, and 30% of the cases closed spontaneously in the later stage of LT, and there were no neurological complications during the overall follow-up period [27]. Werlang et al. expressed the same opinion in a study in 2016, which showed that the presence of asymptomatic PFO did not worsen perioperative outcomes in patients undergoing liver transplantation and there is also no evidence that these patients should undergo surgery or the percutaneous repair of PFO before liver transplantation [28]. In the survey by Flynn et al. [29], the overall incidence of HAT ranged from 0 to 28.1%, and that of PVT ranged from 1.5 to 11.2%. Thrombosis after pediatric liver transplantation occurs as a result of various factors, such as changes in blood flow caused by anastomosis difficulties, narrower blood vessels, anatomical variations, and an increased tendency to form blood clots due to acquired deficiencies in protein S, protein C, and antithrombin [29]. Our findings showed no significant difference in the incidence of HAT or PVT between the two groups. In our study, no ischemic stroke occurred during hospitalization in the two groups, and there were no significant differences in the incidences of perioperative complications and mortality between the two groups. In terms of the previous surveys and our study, the presence of an asymptomatic PFO in children undergoing LDLT may have no clinical implication.

The results of this study showed that the PELD score in children in the PFO group was higher than that in the NoPFO group. However, according to the statistical result, we did not find an increase in mortality associated with the elevated PELD score in the PFO group. The factors influencing the PELD score included the serum bilirubin level, albumin level, INR, age and growth stagnation, among which liver function and the coagulation indexes had the greatest influence and were closely associated with prognoses [30]. In addition, Evelyn et al.‘s study showed that a developed PELD score using preoperative serum sodium, creatinine, and updated PELD components could increase the predictive value of the original PELD score [31]. In the present study, there were no significant differences in preoperative ALT, AST, creatinine and INR levels between the two groups, which may be related to the similar short-term outcomes between the two groups. Although 4 pediatric patients died after LDLT in the PFO group, according to the analysis of the cause of death, there was no relationship between PFO and the postoperative death.

Our study is the first retrospective study performed to investigate the effect of PFO on clinical outcomes during the perioperative period in patients undergoing LDLT. There have been no previous studies that have used such a large sample size. However, this study also had some limitations. First, because children with ESLD may have complications due to esophageal varices, transesophageal echocardiography (TEE) examination could not be carried out in every child in this study, and the diagnosis of PFO may have been missed in a small number of children. Second, since this was a single-center study conducted in China, we are not sure whether the results can be generalized to other ethnic groups. Therefore, on the basis of this single-center retrospective study, further large-sample multicenter observational studies are recommended.

## Conclusions

This study described the experience in our center of the treatment of children with PFO and biliary atresia, all of whom successfully underwent LDLT. No ischemic stroke occurred during hospitalization in both groups. There were no significant differences in the incidence of PRS, postoperative mechanical ventilation time, ICU stay duration, postoperative hospital stay duration, incidences of postoperative complications (AKI, graft dysfunction, HAT and PVT) or the one-year survival rate between the two groups. PFO did not have negative effects on the short-term outcomes of LDLT in children. For children with biliary atresia and PFO, LDLT is safe, and PFO does not increase the incidences of perioperative adverse events or mortality.

## Data Availability

All data generated or analyzed during this study are included in this published article.
